# EEG Microstates as a Signature of Hemispheric Lateralization in Stroke

**DOI:** 10.1007/s10548-023-00967-8

**Published:** 2023-05-17

**Authors:** Maria Rubega, Massimiliano Facca, Vittorio Curci, Giovanni Sparacino, Franco Molteni, Eleonora Guanziroli, Stefano Masiero, Emanuela Formaggio, Alessandra Del Felice

**Affiliations:** 1 Department of Neuroscience, Section of Rehabilitation, University of Padova, Via Giustiniani 3, 35128 Padova, Italy; 2 Padova Neuroscience Center, University of Padova, Via Orus 2, 35131 Padova, Italy; 3 Department of Information Engineering, University of Padova, via Gradenigo 6/b, 35128 Padova, Italy; 4 Villa Beretta Rehabilitation Center, Valduce Hospital, Via Sauro 17, 23845 Costa Masnaga, Lecco Italy; 5 Department of Neuroscience, Section of Neurology, University of Padova, Via Giustiniani 3, 35128 Padova, Italy

**Keywords:** Stroke, EEG, Hemispheric lateralization, Microstates

## Abstract

Stroke recovery trajectories vary substantially. The need for tracking and prognostic biomarkers in stroke is utmost for prognostic and rehabilitative goals: electroencephalography (EEG) advanced signal analysis may provide useful tools toward this aim. EEG microstates quantify changes in configuration of neuronal generators of short-lasting periods of coordinated synchronized communication within large-scale brain networks: this feature is expected to be impaired in stroke. To characterize the spatio-temporal signatures of EEG microstates in stroke survivors in the acute/subacute phase, EEG microstate analysis was performed in 51 first-ever ischemic stroke survivors [(28–82) years, 24 with right hemisphere (RH) lesion] who underwent a resting-state EEG recording in the acute and subacute phase (from 48 h up to 42 days after the event). Microstates were characterized based on 4 parameters: global explained variance (GEV), mean duration, occurrences per second, and percentage of coverage. Wilcoxon Rank Sum tests were performed to compare features of each microstate across the two groups [i.e., left hemisphere (LH) and right hemisphere (RH) stroke survivors]. The canonical microstate map D, characterized by a mostly frontal topography, displayed greater GEV, occurrence per second, and percentage of coverage in LH than in RH stroke survivors (p < 0.05). The EEG microstate map B, with a left-frontal to right-posterior topography, and F, with an occipital-to-frontal topography, exhibited a greater GEV in RH than in LH stroke survivors (p = 0.015). EEG microstates identified specific topographic maps which characterize stroke survivors’ lesioned hemisphere in the acute and early subacute phase. Microstate features offer an additional tool to identify different neural reorganization.

**Supplementary Information:**

The online version contains supplementary material available at 10.1007/s10548-023-00967-8.

## Introduction

Stroke is one of the leading causes of disability burden globally and the main determinant of motor impairment. It affects around 17 million people every year and stroke-induced impairments are expected to increase in the next decades due to aging (Donkor 2018).

Electroencephalography (EEG) is a widely available technique providing a direct measure of brain activity. Quantitative EEG (qEEG), mostly in the frequency domain and from a linear perspective (Bentes et al. 2018), is a widely used method to study cerebral modifications after stroke. Recently, methods of complexity and self-similarity within the information theory framework have been added to the toolbox of EEG analysis in stroke (Rubega et al. 2021). However, to date the topographical richness of EEG, as well as its temporal dimension, have been largely underexplored.

A feasible approach to dissect the spatial-temporal dynamics underlying EEG topographies is provided by EEG microstates, defined as global patterns of spontaneous brain activity showing a topographical stability and a low dimensional structure (Murray et al. 2008). Accordingly, intrinsic brain activity can be explained by a few quasi-stable scalp potential maps lasting for a limited period of time (60−120 ms), which are highly reproducible and consistent across subjects (Michel and Koenig 2018). A four-class microstate topography sequence has been described: class A, characterized by right-frontal to left-posterior activity, class B by left-frontal to right-posterior activity, class C by frontal to occipital activity, and class D by a mainly frontal and less occipital activity than C. The optimal number of clusters, however, should be estimated for each dataset individually using robust optimization criteria, rather than determining a fixed number (Michel and Koenig 2018). These transient topographies are thought to arise from the coordinated activity of millions of neurons across the cortex and have shown to be closely related to the canonical resting-state networks, as identified by functional Magnetic Resonance Imaging (fMRI) (Abreu et al. 2021). Due to the intrinsic low-dimensional structure of EEG microstates, they are considered to be the building blocks of spontaneous brain activity or “atoms of thought”. Coherently, a great effort has been directed towards the study of these quasi-stable topographies in health and disease. To date, EEG microstates analysis has rarely been applied to stroke, describing a different exploration of neural states C and, to a lesser extent, D (Zappasodi et al. 2017). The integrity of the microstate B turned was linked to a better functional outcome (Zappasodi et al. 2017). The changes in microstate dynamics for stroke survivors appear to be state-selective and related to brain dysfunction after stroke and subsequent functional reconfiguration, but no clear correlation with the side of lesion emerged (Hao et al. 2022). In fact, we know that the side of stroke lesion might have an impact on cortical reorganization and recovery (e.g., Molteni et al. 2020; Park et al. 2016; Rubega et al. 2021).

Our hypothesis is that a selective effect of the lesion side may affect spatial-temporal characteristics of microstates.

Here, we investigate the spatial-temporal dynamics of EEG microstates during resting-state in a cohort of first-ever people with stroke (n = 51) recruited during the first 3 months after the event specifically looking for a different prevalence of spatio-temporal microstates dynamics in participants with right and left hemispheric lesions.

## Results and Discussion

**Fig. 1:**
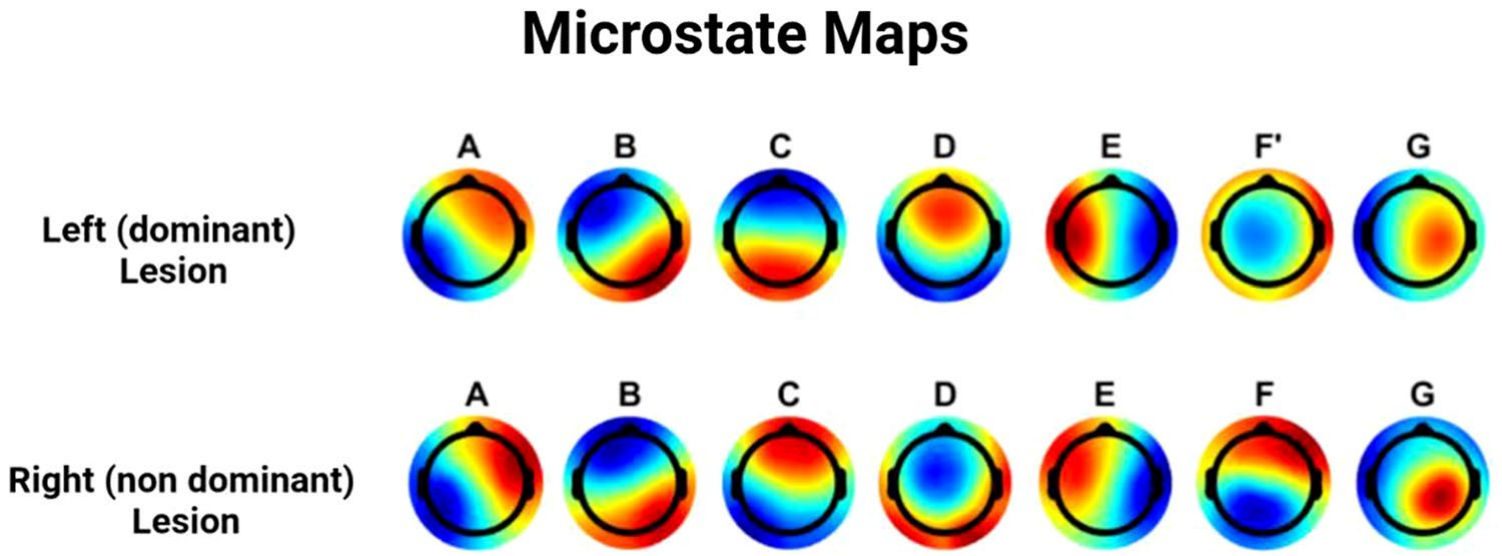
Ordered and named microstate maps of the two groups (left and right hemisphere stroke). Topographies were ordered based on Custo et al. (2017)

51 stroke survivors were recruited [24 with a right hemisphere (RH) lesion (age in interval 29–82 years)]. Maximizing the Global Explained Variance (GEV) and minimising the Cross-Validation Criterion at the single subject level yielded an optimal number of microstate classes equal to 6 for more than half of the stroke survivors, regardless of the specific site of the lesion. Hence, the initial group analysis was performed to extract a number of classes equal to 6. However, after the visual inspection of right and left population-specific scalp topographies and correlation matrices, we decided to further extend the number of classes to 7. The spatial correlation (ρ) of the first five maps (i.e., named A, B, C, D and E) between RH and LH was, respectively, [0.96, 0.96, − 0.91, − 0.9, 0.93]. The 6th map differs between the two groups (i.e., ρ = 0.02). Thus, we decided to extract an additional map (i.e., seven maps in total): (i) to see how the spatial correlation was affected; (ii) and beacuse, based on findings in (Custo et al. 2017), which included 164 subjects during 3–7 min time frame and exploited a meta-criterion of 11 individual optimization criteria to define the number of clusters, seven cluster maps result in optimally describing the data, i.e., explaining the 84.4% of the variance across all subjects (Michel and Koenig 2018). The statistical analysis of microstate features was performed on seven different maps, which were ordered and named as reported in Fig. 1 (ρ = [0.96, 0.96, − 0.91, − 0.9, 0.93, 0.26, 0.91]). Considering the values obtained for the spatial correlation, we decided to name the sixth map differently for the two groups, i.e., F′ and F.

**Fig. 2:**
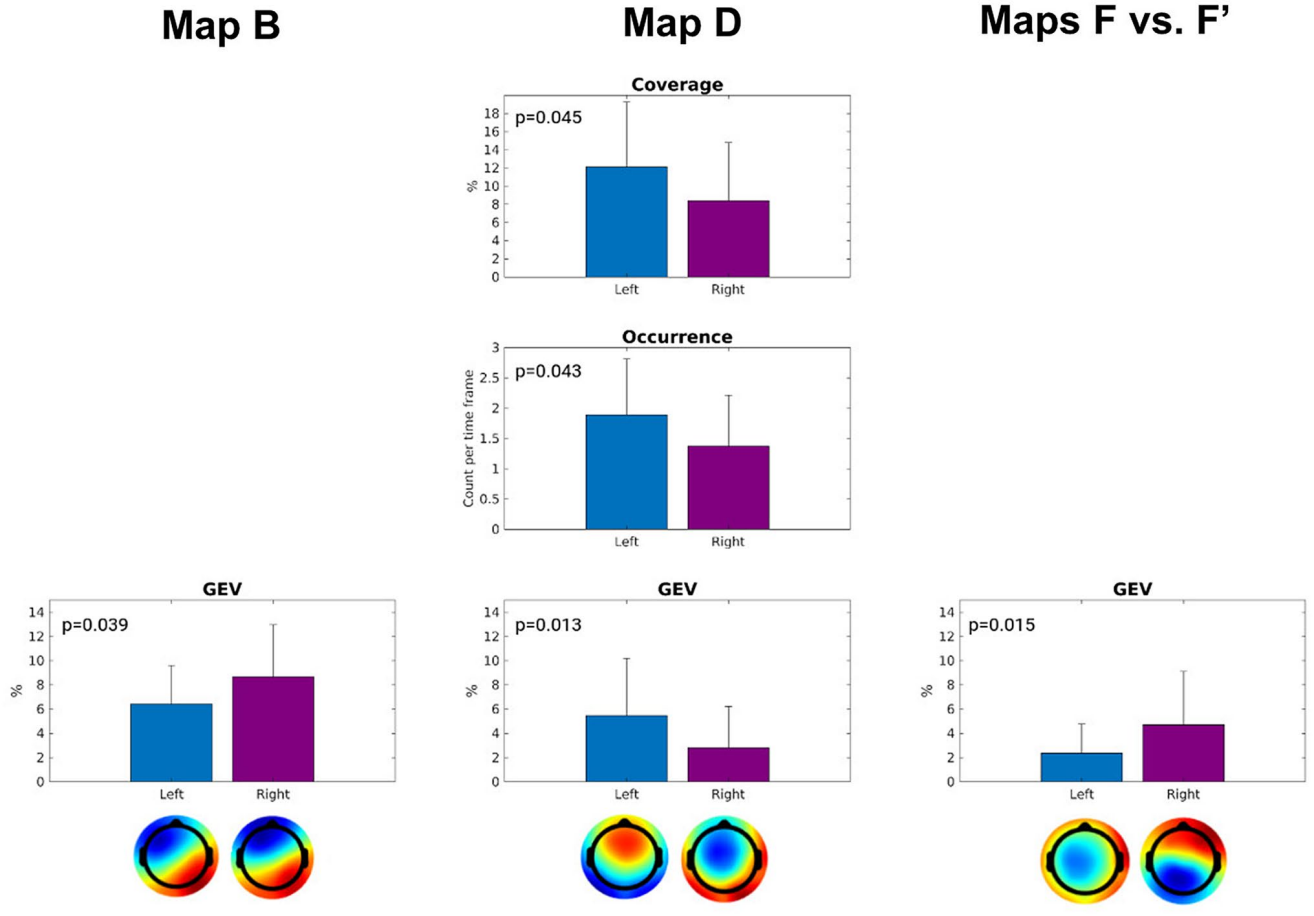
Mean value of microstate features and associated p-values of the difference between LH and RH stroke for microstate classes B, D and F′ vs. F. Error bars indicate the standard deviation

Comparing the microstate features in left lesion vs. right lesion stroke survivors, we obtained statistically significant differences in microstate classes B, D, and F vs F′ (Fig. 2). The GEV of map B was larger in the RH survivors (p = 0.039). GEV, frequency of occurrence per second, and percentage of coverage of microstate class D were significantly lower in RH survivors (all p < 0.05). The GEV of the microstate map F vs. F′ was significantly greater in RH than in LH (p = 0.015).

The EEG microstate analysis provides a feasible approach to study the global state of functional neural networks, as meta-stable scalp potential maps are thought to constitute a proxy of how the global coordination of neurons changes over time. Hence, microstates provide a window into neural dynamics beyond local scale approaches. The global nature of microstates is thus well suited to signal aberrations of neural communication in stroke, a disorder known to have a massive impact on the brain across different spatial and temporal scales. We predicted that microstates features would differ as a function of the side of the lesion in right-handed stroke survivors. In agreement with this hypothesis, although we are aware that using different group template maps might increase the number of false positive results in the statistics of the extracted features, we found a significant difference in microstate features based on stroke localization for microstates classes B, D and F vs F′. A significant finding was that microstate class F vs. F′ apparently distinguished right vs. left stroke lesions as its spatial representation differs and GEV is significantly larger in stroke survivors suffering from a stroke lesion in the right hemisphere. Map B, characterized by a non-symmetric activity as map F, also has a significantly higher GEV in right vs. left stroke lesions. This hypothesis needs to be also confirmed in an extended dataset, which would allow a proper stratification of participants. Investigating microstates representation in a homogeneous group will allow the use of the same group templates and overcome the main issue of this work in the statistics of the extracted features. Ischemic lesions are highly heterogeneous in localization, causing functional disconnections which may differently impact also on microstates representation. Hemispheric specificity is a well known phenomenon in upper and lower limb motor control in able bodied subjects as well as in stroke survivors (Molteni et al. 2020). In people with stroke, we observed a better functional outcome in persons with non-dominant lesions, possibly related to a different motor reserve in the two hemispheres (Molteni et al. 2020). Our results add on previous research highlighting the role of the lesioned hemisphere in determining features of global scale EEG topographies. Microstate features offer an additional tool to identify different neural reorganization in the two hemispheres. For example, microstate segmentation could be exploited to subdivide EEG resting-state to investigate specific effective connectivity networks.

### Supplementary Information

Below is the link to the electronic supplementary material.

Supplementary file1 (DOCX 15 kb)

Supplementary file2 (XLSX 12 kb)
