# Supplementary material for: EEG Microstates as a Signature of Hemispheric Lateralization in Stroke
Source: Brain Topogr. 2023 May 17;37(3):475–8. doi: 10.1007/s10548-023-00967-8 (PMC10191079; doi:10.1007/s10548-023-00967-8)
Supplement: Supplementary file 1 — Supplementary file1 (DOCX 15 kb) [file 10548_2023_967_MOESM1_ESM.docx]

**(ONLINE SUPPLEMENT)**

**Materials and Methods**

**Participants**

51 stroke survivors were recruited in two different hospitals: Dataset 1, constituted by 16 participants admitted to the Stroke Unit of Padua Teaching Hospital during hyperacute (<48 h) or acute phase (∼1st week after stroke) and Dataset 2 of 35 participants monitored at the Neurorehabilitation Unit of Villa Beretta Valduce Hospital during the early subacute phase (8 to 32 days after stroke). Dataset 1 comprised 16 right-handed subjects, 6 with a right hemisphere lesion (56-81 years, 33% female) and 10 with a left hemisphere lesion (30-83 years, 10% female). Dataset 2 consisted of 35 right-handed participants, 18 with a right hemisphere lesion (38-80 years, 47% female) and 17 with a left hemisphere lesion (43-81 years, 39% female).

**EEG recording**

An EEG resting-state recording with eyes-open (EO) was acquired for each participant. EEG in Dataset 1 consist of a 5-min resting-state recording with a 30-channels EEG cap (BrainAmp 32MRplus, BrainProducts GmbH, Munich, Germany, the reference was positioned between Fz and Cz and ground anterior to Fz); in Dataset 2 the eyes open resting-state EEG lasted 10 min and was recorded through a 60-channels EEG cap (Compumedics Neuroscan, Compumedics, NC, USA, the reference was positioned between Fz and Cz and the ground anterior to Fz). We refer the reader to Rubega et al. (2021) for further details of the datasets.

**EEG pre-processing**

The data were preprocessed in MATLAB R2019b (MathWorks, Natick, MA, USA) with a custom-made script based on EEGLAB toolbox. The EEG was band-pass filtered between 1 and 30 Hz with a zero-phase filter to remove slow drifts and 50 Hz power line noise. The optimal Chebyshev finite impulse response filters were designed using Parks–McClellan algorithm, the order was customized to minimize the error in the pass and stop bands. Subsequently, the signal was downsampled at 500 Hz and eye blinks, eye movements, and cardiac activity were removed with Independent Component Analysis (FastICA algorithm) as implemented in EEGLAB based on power spectrum, topography, and time course. Lastly, data were re-referenced to the average reference. The EEG data of Dataset 2 were spatially down-sampled to match the spatial resolution of Dataset 1 (30 active channels).

**Microstate analysis**

Following the procedure described by Murry et al. (2008) and for each participant separately, the Global Field Power (GFP) time series was computed as the standard deviation of the EEG channels across the scalp. Next, local maxima along the GFP time series were extracted and submitted to a modified polarity-invariant k-means clustering algorithm to identify a low-dimensional set of predominant scalp topographies. The number of clusters was liberally set between 4 (standard) and 7 (as in Custo et al, 2017). The subject-specific optimal number of classes was selected by maximizing the goodness of fit and minimizing the complexity of the model, indexed by the Global Explained Variance (GEV) and cross-validation criterion, respectively. As the optimal number corresponded to 6 for more than half of subjects, subsequent analyses were performed accordingly. To identify the population level microstates, stroke survivors were divided in two groups based on the side of the brain lesion and EEG maps at the peaks of the GFP were concatenated across the subjects belonging to each group. Subsequently, a modified k-means clustering algorithm was implemented to obtain the population level maps by setting the number of clusters to be equal to 6. After the visual inspection of right and left population-specific scalp topographies and correlation matrices (i.e., there was a high correlation between the first 5 maps whereas the 6th map differed between the two groups), we decided to further extend the number of classes to 7 in order to increase the map comparability between the two groups. We computed again the population level maps by setting the number of clusters equal to 7 for both groups. Based on the Global Map Dissimilarity (GMD) index, a backfitting procedure was implemented to assign each subject-specific EEG sample to the group-level map which minimized the dissimilarity between the two topographies. Subsequently, each subject-specific microstates time series was temporally smoothed by removing topographies lasting for less than 30 ms and reassigning them based on the similarity to the topographical neighborhood (previous and subsequent microstates lasting more than 30 ms). For each microstate map, 4 class-specific parameters were quantified: Global Explained Variance (GEV), occurrence per second, mean duration and percentage of coverage. Due to the non-gaussianity of microstate features, we performed two-sided Wilcoxon Rank Sum tests (p_value_ < 0.05) to compare the features of each microstate class among the two groups: right hemisphere stroke and left hemisphere stroke survivors. Considering the sample size and the exploratory nature of the study, no corrections for multiple comparisons were performed in hypothesis testing.

**References**

[1] Custo, A., Van De Ville, D., Wells, W. M., Tomescu, M. I., Brunet, D., & Michel, C. M. (2017). Electroencephalographic Resting-State Networks: Source Localization of Microstates. *Brain connectivity*, *7*(10), 671–682. <https://doi.org/10.1089/brain.2016.0476>

[2] Murray, M.M., Brunet, D. & Michel, C.M. (2008) Topographic ERP Analyses: A Step-by-Step Tutorial Review. Brain Topogr (20) 249–264. doi: <https://doi.org/10.1007/s10548-008-0054-5>
